# The relationship between self-efficacy and perceived academic achievement among college students: the chain mediation roles of time management and psychological resilience

**DOI:** 10.3389/fpsyg.2026.1852212

**Published:** 2026-09-14

**Authors:** Li Gao, Wanchun Wang

**Affiliations:** School of Marxism, Chuzhou University, Chuzhou, Anhui, China

**Keywords:** college students, perceived academic achievement, psychological resilience, self-efficacy, time management

## Abstract

**Background:**

This study attempts to investigate the link between self-efficacy and perceived academic achievement among university students, with an emphasis on the mediating roles of time management and psychological resilience.

**Methods:**

This study targeted college students from 6 universities in Anhui Province, utilizing a questionnaire-based approach for data collection. Instruments such as the General Self-Efficacy Scale (GSES), Academic Achievement Scale, Time Management Questionnaire (TMQ), and the 10-item Connor-Davidson Resilience Scale (CD-RISC-10) were employed, resulting in 818 valid responses. Partial Least Squares Structural Equation Modeling (PLS-SEM) was used to analyze the association between self-efficacy and perceived academic achievement, besides the serial mediating roles of time management and psychological resilience.

**Results:**

(1) University students' self-efficacy is significantly and positively associated with perceived academic achievement. (2) time management and psychological resilience play a sequential mediating role between self-efficacy and perceived academic achievement. The structural model explained 59.1% of the variance in perceived academic achievement.

**Conclusion:**

This study not only expands our knowledge of how self-efficacy, time management, and psychological resilience linked to perceived academic achievement, but also provides important practical guidance for enhancing students' academic performance. The findings emphasize that improving self-efficacy, optimizing time management strategies, and fostering psychological resilience are crucial pathways to enhancing perceived academic achievement. Therefore, educators and relevant policymakers should implement more targeted interventions in academic support and psychological development to effectively improve students' academic performance.

## Introduction

Perceived academic achievement has long been an important topic in educational psychology. It reflects students' subjective evaluations of their academic performance and learning outcomes and is closely associated with their career development and competitiveness (Eakman et al., 2019; Luo et al., 2023). With the global expansion of higher education, the competitive pressure on university students regarding academic development and future careers has increased, and the issue of perceived academic achievement has drawn increasing attention in educational research (Alyahyan and Düstegör, 2020; Xu, 2024). In this context, how to enhance perceived academic achievement has become an urgent issue for contemporary university students.

Research indicates that perceived academic achievement is shaped by various factors, including individual characteristics (such as psychological resilience, self-efficacy, cognitive ability, and learning motivation), behavioral factors (such as time management and learning strategies), and social environmental factors (including family, school, and societal contexts) (Halat et al., 2023; Shengyao et al., 2024; Tompsett and Knoester, 2023; Xu, 2024). Although learning strategies, learning motivation, and social support are also associated with perceived academic achievement, this study focuses on time management and psychological resilience. These two factors represent complementary mechanisms of behavioral self-regulation and psychological adaptation through which self-efficacy is associated with academic outcomes. Time management reflects students' ability to plan academic tasks and allocate time resources, while psychological resilience reflects their ability to maintain or regain a positive learning state when facing academic stress and setbacks (Ali et al., 2024; Garcia-Martinez et al., 2022). Effective time management may also be associated with greater psychological resilience by reducing task accumulation and strengthening students' sense of control. Thus, these two factors may not only separately link self-efficacy to perceived academic achievement but also form a sequential pathway. Social Cognitive Theory (SCT) holds that individual cognition, behavior, and environmental conditions are dynamically interrelated. The theory also suggests that individuals' cognitive judgments and self-regulation are associated with their behavior and related outcomes (Bandura, 1986). In this study, self-efficacy represents students' cognitive judgments about their ability to complete tasks. Time management reflects behavioral self-regulation, while psychological resilience represents the adaptive psychological resources that students draw on when facing academic stress and setbacks. Self-efficacy is closely associated with students perceived academic achievement. It may also be indirectly related to academic outcomes through self-regulatory behaviors and persistence when students face difficulties (Hanham et al., 2021). However, the majority of research to date has focused on the direct link between self-efficacy and perceived academic achievement. Less attention has been given to the separate and sequential mediating roles of time management and psychological resilience. Thus, examining time management and psychological resilience together may help clarify the potential pathways linking self-efficacy to perceived academic achievement.

### Self-efficacy and perceived academic achievement

Self-efficacy, a key psychological concept, denotes a person's confidence in their capacity to successfully finish particular activities or overcome obstacles (Liu et al., 2024; Popa-Velea et al., 2021; Zheng et al., 2023). Unlike academic self-efficacy, which focuses on specific courses or academic tasks, general self-efficacy shows views about one's capabilities across different contexts. This study focuses on general self-efficacy because its cross-contextual nature is more consistent with the conceptual features of time management and psychological resilience. All three constructs involve self-regulation and adaptation beyond specific academic tasks. Academic achievement can be measured using objective indicators, like course grades and grade point average (GPA), or through students' subjective evaluations of their learning outcomes. Perceived academic achievement, or students' subjective assessments of their overall academic performance, learning progress, and attainment of academic goals. We measured this construct using a self-report scale. It reflects students' perceived learning outcomes rather than objective academic performance, such as course grades or GPA. Thus, perceived academic achievement is conceptually related to, but distinct from, objective academic achievement.

Previous research has demonstrated that self-efficacy is strongly linked to perceived academic achievement (Huang and Wang, 2023; Meng and Zhang, 2023; Widowati et al., 2023). For university students, higher self-efficacy not only enhances learning motivation but also enables them to demonstrate stronger adaptability and problem-solving skills when facing academic challenges (Basileo et al., 2024; Chen, 2024; Honicke et al., 2023). According to Meng and Zhang (2023), students who have higher self-efficacy are more adept at controlling their emotions and adjusting strategies when faced with difficulties, leading to greater learning engagement and improved perceived academic achievement. Furthermore, Basileo et al. (2024) noted that students who have high self-efficacy are more likely to have stronger learning motivation and are more capable of setting realistic academic goals, thereby improving their academic performance. Therefore, self-efficacy is a core indicator of perceived academic achievement and is essential for overcoming obstacles in the classroom and achieving academic goals.

### The mediating role of time management

Time management is the capacity of a person to efficiently plan, allocate, and utilize time within a constrained timeframe to accomplish specific objectives (Aeon et al., 2021; Au et al., 2023; Sekizler et al., 2022; Wolters and Brady, 2021). Effective time management increases learning effectiveness and generally improves the general quality of life for university students (Ali et al., 2024). As the educational environment and societal demands evolve, time management has increasingly become one of the key factors for improving perceived academic achievement. Effective time management helps students to balance study tasks and rest periods, thus enhancing learning efficiency, reducing anxiety and stress, and ultimately improving perceived academic achievement (Adams and Blair, 2019; Trentepohl et al., 2022; Waldeyer et al., 2022). Research by Trentepohl et al. (2022) indicated that good time management improves students' learning performance, reduces academic procrastination, and significantly enhances perceived academic achievement. Furthermore, Waldeyer et al. (2022) suggested that time management helps students achieve academic goals and strengthens their confidence in completing tasks through proper planning, and this positive feedback further boosts their perceived academic achievement. Therefore, time management is essential to raising university students perceived academic achievement.

Many studies also demonstrate the close link between self-efficacy and time management. Students that have high self-efficacy tend to excel in time management, as they trust their capacity to complete assignments and demonstrate effective organizational and planning skills (Bargmann and Kauffeld, 2023; Dong et al., 2024; Ersoy and Peker, 2020). Conversely, students with low self-efficacy may feel helpless when faced with complex tasks, leading to procrastination and ineffective time management behaviors. In addition, the study by Ersoy and Peker (2020) showed that students with high self-efficacy generally perform better on academic assignments. They demonstrate higher self-discipline and initiative in time management and are better at regulating their emotions under pressure, thus reducing procrastination. Therefore, time management directly relates to perceived academic achievement and mediates the link between self-efficacy and perceived academic achievement. Specifically, students with high self-efficacy effectively plan study assignments thru good time management, which enhances perceived academic achievement. Research conducted by Dong et al. (2024) revealed that students with higher levels of self-efficacy are prone to develop well-structured study plans and consistently follow them. According to SCT, self-efficacy, a measure of one's positive attitudes about one's abilities, may be linked to better time management, which may then be linked to higher perceived academic accomplishment (Bandura, 1986). Thus, time management plays an important mediating role between self-efficacy and perceived academic achievement.

### The mediating role of psychological resilience

Psychological resilience describes the ability to adopt a positive, adaptive mindset and effectively navigate challenges, stress, and adversity (Gu and Xue, 2022; Shen et al., 2024; Yarayan et al., 2024; Zhang et al., 2024). Psychological resilience is not a completely stable individual trait. Instead, it is a dynamic process through which individuals draw on internal and external resources to regulate themselves and adapt positively in specific situations (Gorczynski and Webb, 2022; Masten, 2014; Ungar, 2021). From the perspective of SCT, psychological resilience can be viewed as an adaptive psychological resource. It reflects students' ability to adjust their psychological state and maintain engagement in learning activities when facing academic stress and setbacks (Bandura, 1986). Studies have identified a significant positive link between psychological resilience and perceived academic achievement among university students (Garcia-Martinez et al., 2022; Gorczynski and Webb, 2022; Tekin, 2024). Research by Gorczynski and Webb (2022) indicated that students with higher psychological resilience are better able to manage academic stress and challenges, maintaining calmness and confidence, adopting positive coping strategies, and adjusting their learning methods, which leads to higher perceived academic achievement. Similarly, Garcia-Martinez et al. (2022) suggested that resilient students exhibit strong psychological resistance to stress, enabling them to learn from failure, modify their tactics in a timely manner, and ultimately enhance their academic performance.

Research shows that individuals with higher self-efficacy are more inclined to strengthen their psychological resilience through effective positive emotional regulation (Li et al., 2023; Qin et al., 2023). Li et al. (2023) indicated that self-efficacy, as a key psychological resource, helps students form an accurate perception and understanding of their abilities, enabling them to face challenges positively rather than succumb to negative emotions, thereby demonstrating higher psychological resilience in situations of difficulties. Furthermore, Qin et al. (2023) suggested that students with high self-efficacy are more confident in overcoming academic obstacles, and this belief significantly boosts their psychological resilience, which further enhances their perceived academic achievement. Therefore, psychological resilience is directly associated with perceived academic achievement and mediates the link between self-efficacy and perceived academic achievement. Specifically, students with strong self-efficacy improve their psychological resilience, which enables them to better handle academic pressures, failures, and challenges, accumulate success experiences and confidence, and create favorable conditions for improving their academic performance.

### The chain mediation role of time management and psychological resilience

In research on perceived academic achievement, self-efficacy, time management, and psychological resilience may form a sequential pathway. Higher self-efficacy among college students has been linked to improved time management and psychological resilience, according to earlier research. They also show increased self-assurance and adaptability when confronted with academic tasks and obstacles (Au et al., 2023; Dong et al., 2024; Li et al., 2023). At the same time, efficient time management is linked to greater psychological resilience because organizing academic tasks appropriately may reduce academic stress and strengthen students' sense of control over the learning process (Antoniadou and Antoniadis, 2024; Förster et al., 2023; Peykar et al., 2023). SCT provides a theoretical basis for this sequential relationship. The theory suggests that individuals' cognitive beliefs are associated with their behavior and adaptive outcomes through self-regulatory processes (Bandura, 1986). As a core cognitive belief, self-efficacy may be related to students' greater willingness to set goals, plan their learning, and maintain engagement. These tendencies may, in turn, be associated with more effective time management (Dong et al., 2024). Effective time management may also reduce task accumulation and time pressure and strengthen students' sense of control when dealing with academic difficulties. Thus, it may be associated with greater psychological resilience (Antoniadou and Antoniadis, 2024; Knezevic and Polak, 2024). Previous serial mediation studies have also shown that students' cognitive beliefs, self-regulatory characteristics, and adaptive psychological resources may be sequentially associated with academic behaviors or outcomes (Shao and Kang, 2022). Although these studies examined variables that differ from those in the present study, their results provide a sequential psychological process to comprehend academic outcomes. Thus, this study proposes that self-efficacy may first be associated with better time management, which may then be associated with greater psychological resilience and, ultimately, higher perceived academic achievement.

### Current research

There are still a lot of gaps in the literature, despite the growing amount of research on academic stress among college students. Most existing research has primarily concentrated on the direct association between self-efficacy and perceived academic achievement, with limited attention to the indirect links between self-efficacy and perceived academic achievement through time management and psychological resilience. Based on SCT, this study conceptualizes self-efficacy, time management, and psychological resilience as personal cognition, behavioral self-regulation, and an adaptive psychological resource, respectively. It examines the separate and sequential mediating roles of time management and psychological resilience in the link between self-efficacy and perceived academic achievement. By developing this integrated model, the study intends to offer a more systematic understanding of the potential pathways linking self-efficacy to perceived academic achievement. It also seeks to provide a theoretical basis for educational strategies that support college students perceived academic achievement. In conclusion, this study presents the research model illustrated in Figure 1 and evaluates the following hypotheses:

H1: University students' self-efficacy and perceived academic achievement are significantly positively correlated.
H2: Time management mediates the link between self-efficacy and perceived academic achievement.
H3: Psychological resilience mediates the link between self-efficacy and perceived academic achievement.
H4: Time management and psychological resilience jointly mediate the link between self-efficacy and perceived academic achievement.

**Figure 1:**
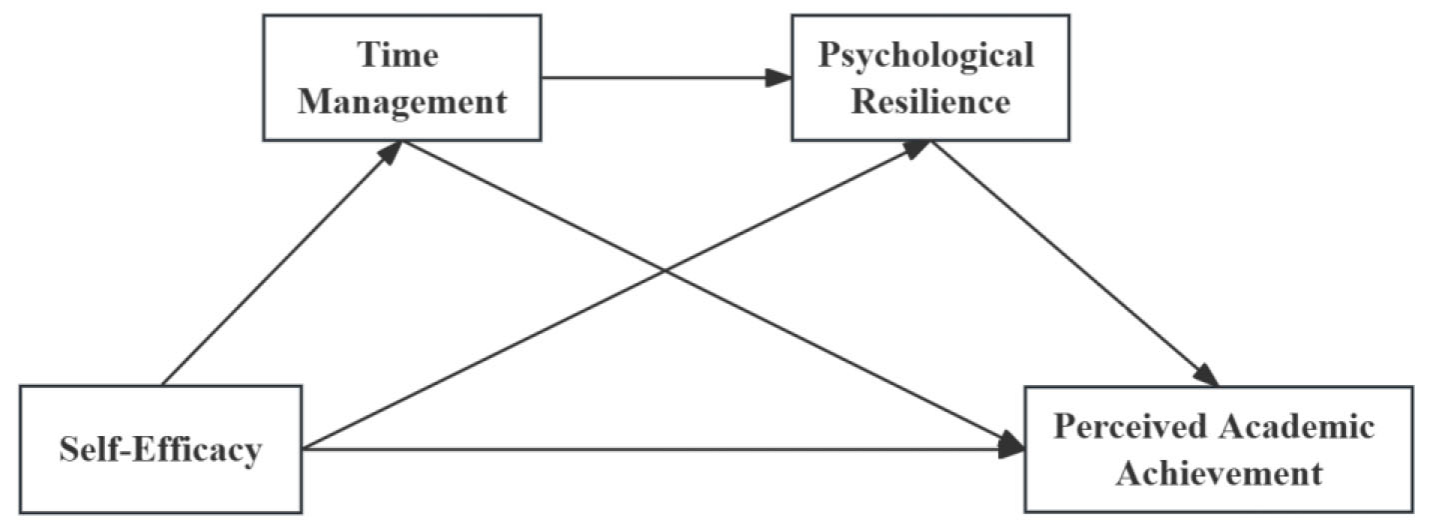
Research model.

## Method

### Data collection

We conducted this study in Anhui Province, China, from February to March 2026. We used convenience sampling and selected six universities based on the research team's access to potential participants and the universities' willingness to participate. The six universities were University of Science and Technology of China, ANHUI UNIVERSITY OF CHINESE MEDICINE, Anhui Polytechnic University, BENGBU MEDICAL UNIVERSITY, ANHUI UNIVERSITY OF SCIENCE and TECHNOLOGY, and FUYANG NORMAL UNIVERSITY. With support from the participating universities or departments, the research team recruited eligible full-time undergraduate students. We distributed the questionnaire online through Wenjuanxing (https://www.wjx.cn/). Teachers or student representatives at each university shared the questionnaire link with potential participants through university communication channels, email, and student social media groups. After reviewing the study materials and giving their informed consent, students willingly took part.

To ascertain the minimal necessary sample size, we performed an a priori power analysis using G^*^Power 3.1 prior to data collection. With a significance level of α = 0.05, statistical power of 1 – β = 0.95, a medium effect size of *f* ^2^ = 0.15, and a maximum of eight predictors, the analysis demonstrated that a minimum required sample size of 160 participants. We collected 832 questionnaires. After excluding questionnaires with missing data or inconsistent responses, we retained 818 valid questionnaires, resulting in a valid response percentage of 98.32%. The resulting sample size offered sufficient statistical power for PLS-SEM model estimation and hypothesis testing, and it was significantly bigger than the minimal sample size required by the a priori power analysis. The sample included 127 students from University of Science and Technology of China, 134 from ANHUI UNIVERSITY OF CHINESE MEDICINE, 129 from Anhui Polytechnic University, 143 from BENGBU MEDICAL UNIVERSITY, 149 from ANHUI UNIVERSITY OF SCIENCE & TECHNOLOGY, and 136 from FUYANG NORMAL UNIVERSITY, for a total of 818 students. Of the valid participants, 245 were male (29.95%) and 573 were female (70.05%). Table 1 presents the detailed demographic characteristics and sample distribution across the six universities.

**Table 1:** Demographic Information.

Demographic characteristics	Category	Quantity	Percentage (%)
Gender	Male	245	29.95%
	Female	573	70.05%
Grade	Freshman	569	69.56%
	Sophomore	83	10.15%
	Junior	143	17.48%
	Senior	23	2.81%
Major	Science and Engineering	221	27.02%
	Humanities and Social Sciences	254	31.05%
	Medicine	11	1.34%
	Arts	151	18.46%
	Others	181	22.13%
Hometown	Rural	604	73.84%
	Urban	214	26.16%
Only Child	Yes	144	17.60%
	No	674	82.40%

### Measurement instruments

The survey's five-point Likert scale, which goes from 1 to 5, allowed respondents to give precise answers depending on their own experiences. The content and structure of the items in this study were not altered in order to preserve consistency with the original scale. We analyzed the General Self-Efficacy Scale and the Psychological Resilience Scale as unidimensional measures. Although the Perceived Academic Achievement Scale and the Time Management Scale include multiple dimensions, this study focused on their overall constructs. Therefore, we did not examine their dimensions as separate variables in the path analysis. For the descriptive statistics and correlation analyses, we calculated the mean score across all items of each scale to represent the overall level of the corresponding construct. We assigned equal weight to all items, and higher mean scores indicated higher overall levels of the corresponding constructs. In the PLS-SEM analysis, we did not use dimension-level mean scores or overall scale mean scores as observed indicators. Instead, we included all items from each scale as direct indicators of the corresponding overall construct. We tested all paths in the structural model at the overall construct level.

### Self-efficacy

This study employs the GSES developed by Zhang and Schwarzer (1995). There are ten items on the scale. A higher score denotes a greater degree of self-efficacy among university students. The scale demonstrated strong internal consistency and convergent validity, with a Cronbach's α of 0.931, a composite reliability (CR) of 0.941, and an average variance extracted (AVE) of 0.617.

### Perceived academic achievement

This study utilized the Academic Achievement Scale created by Feiyan et al. (2011) to assess students' perceived academic achievement through self-reports rather than actual course grades, examination scores, or grade point averages provided by their universities. Therefore, this study refers to this variable as perceived academic achievement. The scale includes 19 items organized into four dimensions: learning dedication, learning performance, interpersonal promotion, and overall performance. Representative items include “The extent to which my completion of academic and work tasks meets my teachers' requirements” and “Overall, how my academic performance compares with that of my classmates.” Participants rated each item according to their actual learning experiences; a higher score reflects a greater perceived academic achievement among the students. The scale demonstrated strong internal consistency and convergent validity, with a Cronbach's α of 0.956, an overall CR of 0.961, and an overall AVE of 0.568.

### Time management

The TMQ created by Huang and Zhan (2001) was used in this investigation. The 44 items on the scale are divided into three categories: Time Value Perception (15 items), Time Monitoring Beliefs (19 items), and Time Efficacy Perception (10 items). Better time management skills are indicated by higher scores on this scale. The scale demonstrated strong internal consistency and convergent validity, with a Cronbach's α of 0.977, an overall CR of 0.978, and an overall AVE of 0.502.

### Psychological resilience

The CD-RISC-10 utilized in this investigation was created by Campbell-Sills and Stein (2007), consists 10 items. Higher scores are indicative of greater levels of psychological resilience among university students. The scale demonstrated strong internal consistency and convergent validity, with a Cronbach's α of 0.909, a CR of 0.925, and an AVE of 0.554.

## Data analysis

We performed PLS-SEM and assessed the measurement and structural models using SmartPLS 4.0. We selected PLS-SEM because this variance-based method is suitable for assessing latent variable measurement quality, structural paths, explanatory power, and predictive relevance. It also emphasizes prediction-oriented model evaluation (Hair et al., 2022; Shmueli et al., 2019). First, we used outer loadings, Cronbach's α, CR, AVE, and the heterotrait–monotrait ratio (HTMT) to evaluate the measurement model's reliability, convergent validity, and discriminant validity. Next, we used path coefficients, coefficients of determination (R^2^), and predictive relevance (Q^2^) to assess the structural model. The statistical significance of the path coefficients and indirect effects was tested using 5,000 bootstrap resamples, and bias-corrected and accelerated 95% confidence intervals (BCa 95% CIs) were published.

## Results

### Descriptive and correlation analysis

According to Pearson's correlation analysis, self-efficacy was significantly positively linked to perceived academic achievement (*r* = 0.707, *p* < 0.001), time management (*r* = 0.529, *p* < 0.001), and psychological resilience (*r* = 0.557, *p* < 0.001). perceived academic achievement was also significantly positively linked to time management (*r* = 0.567, *p* < 0.001) and psychological resilience (*r* = 0.594, *p* < 0.001). Additionally, a strong significant positive link was found between time management and resilience (*r* = 0.660, *p* < 0.001) (Table 2).

**Table 2:** Means, Standard deviations, and correlation analysis (r) of Variables.

Variables	M ±SD	SE	PAA	TM	PR
SE	3.179 ± 0.743	1			
PAA	3.405 ± 0.736	0.707^***^	1		
TM	3.697 ± 0.545	0.529^***^	0.567^***^	1	
PR	3.503 ± 0.626	0.557^***^	0.594^***^	0.660^***^	1

*N* = 818, ^***^p < 0.001, SE, self-efficacy, PAA, perceived academic achievement, TM, time management, PR, psychological resilience.

### Common Method Bias (CMB)

Since self-reports were utilized to quantify every variable, we evaluated CMB using Harman's single-factor test. Nine factors with eigenvalues larger than 1 were found by the analysis. 39.703% of the variance was explained by the first factor, which was less than the 40% cutoff. Thus, there was no indication that CMB was a significant issue in our investigation.

### Measurement model

Systematic validity and reliability testing was used to assess the measurement model. According to the reliability analysis, every factor loading was higher than the 0.70 cutoff. Furthermore, the CR values for self-efficacy, perceived academic achievement, time management, and psychological resilience were 0.941, 0.961, 0.978, and 0.925, as exceeding the minimum standard of 0.70, which indicates strong internal consistency. The AVE values for all latent variables (self-efficacy = 0.617, perceived academic achievement = 0.568, time management = 0.502, and psychological resilience = 0.554) exceeded the threshold of 0.50, aligning with the standards proposed by Henseler et al. (2015). The Fornell-Larcker criterion was used to evaluate discriminant validity, and the results showed that each latent variable's square root of the AVE was higher than its correlations with other constructs (see Table 3), offering additional proof of the model's discriminant validity. Unlike the Pearson correlations in Table 2, which were calculated from the observed scale scores, the off-diagonal values in Table 3 are latent-variable correlations estimated by the PLS-SEM measurement model. Therefore, the correlation coefficients may differ slightly between the two tables.

**Table 3:** Fornell-Larcker criterion.

Variables	PAA	PR	SE	TM
PAA	***0.753***			
PR	0.612	***0.744***		
SE	0.716	0.564	***0.786***	
TM	0.580	0.662	0.533	***0.709***

The square root of each construct's AVE is shown in bold italics on the diagonal.

In order to assess the links between constructs and determine discriminant validity, the HTMT is utilized. Gefen et al. (2011) cautioned that values exceeding 0.85 could indicate a risk of construct overlap. The HTMT criterion was used in this work to assess discriminant validity. The results showed that all HTMT between constructs were less than 0.85 (see Table 4), indicating high discriminant validity and offering a solid basis for further model analysis.

**Table 4:** Discriminant validity (HTMT Criterion).

Variables	PAA	PR	SE	TM
PAA				
PR	0.650			
SE	0.755	0.608		
TM	0.596	0.699	0.556	

### Structural model

The structural model analysis employed several metrics to assess the model's reliability and explanatory power, including multicollinearity assessment, path coefficient significance testing, and the R^2^. These complementary indicators provide a comprehensive evaluation of the model's robustness and predictive capability.

### Multicollinearity testing

Variance Inflation Factor (VIF) were computed to evaluate multicollinearity. According to the results, multicollinearity was not a problem because all of the latent variables' VIF were below the crucial threshold of 3.3 (see Table 5).

**Table 5:** Collinearity test of the structural model.

Variables	PAA	PR	SE	TM
PAA				
PR	2.001			
SE	1.571	1.396		1.000
TM	1.905	1.396		

### Path Hypothesis testing

The results confirming that all hypotheses were effectively supported (Table 6). Among the factors linked to perceived academic achievement, self-efficacy (β = 0.505, *t* =15.935, *p* < 0.001) had the most significant relationships, followed by psychological resilience (β = 0.215, *t* = 5.791, *p* < 0.001) and time management (β = 0.168, *t* = 4.464, *p* < 0.001). Self-efficacy was significantly and positively linked to both time management (β = 0.533, *t* = 17.689, *p* < 0.001) and psychological resilience (β = 0.296, *t* = 7.941, *p* < 0.001). Additionally, time management was significantly and positively linked to psychological resilience (β = 0.504, *t* = 12.196, *p* < 0.001).

**Table 6:** Hypothesis testing results.

Variables	β	*BCa 95% CI LL*	*BCa 95% CI UL*	*t*	*p*	Results
SE → PAA	0.505	0.444	0.566	15.935	< 0.001	Supported
SE → TM	0.533	0.472	0.590	17.689	< 0.001	Supported
SE → PR	0.296	0.223	0.368	7.941	< 0.001	Supported
PR → PAA	0.215	0.143	0.289	5.791	< 0.001	Supported
TM → PAA	0.168	0.092	0.239	4.464	< 0.001	Supported
TM → PR	0.504	0.422	0.583	12.196	< 0.001	Supported

### Explanatory power and predictive relevance

*R*^2^ values are categorized as strong (0.67), moderate (0.33), and weak (0.19) by Chin (1998). R^2^ for perceived academic achievement and psychological resilience were 0.591 and 0.500, respectively, indicating moderate to substantial explanatory power (Table 7). R^2^ for time management was 0.284, denoting weak to moderate explanatory power. The model's good predictive validity and explanatory power are confirmed by the fact that all Q^2^ values are larger than 0.

**Table 7:** Prediction validity and prediction correlation.

Variables	*R* ^2^	$R_{\text{Adjusted}}^{2}$	Q^2^
PAA	0.591	0.589	0.324
TM	0.284	0.283	0.141
PR	0.500	0.499	0.273

### Mediation analysis

We used 5,000 bootstrap resamples to test the indirect effects and reported bias-corrected 95% confidence intervals. The results demonstrate that the link between self-efficacy and perceived academic achievement is significantly mediated by time management and psychological resilience (Table 8). Moreover, time management and psychological resilience serially mediated the link between self-efficacy and perceived academic achievement. This finding offers a fresh viewpoint on how resource management strategies and psychological factors relate to self-efficacy and perceived academic achievement.

**Table 8:** Mediation analysis.

Relationship	β	*t*	*p*	*BCa 95% CI LL*	*BCa 95% CI UL*	Mediation Type
SE → TM → PAA	0.090	4.235	< 0.001	0.048	0.131	CPM
SE → PR → PAA	0.064	4.974	< 0.001	0.040	0.090	CPM
SE → TM → PR → PAA	0.058	4.689	< 0.001	0.036	0.085	CPM

CPM, Complementary Partial Mediation.

## Discussion

This study examines the direct and indirect associations between college students' self-efficacy and perceived academic achievement, with a specific focus on the mediating roles of time management and psychological resilience. By applying PLS-SEM, the study conducted a thorough examination of how self-efficacy, time management, and psychological resilience relate to college students perceived academic achievement. The results confirm all hypotheses, indicating that self-efficacy is positively linked to perceived academic achievement and indirectly relates to it through time management and psychological resilience. When combined, these variables account for 59.1% of the variation in perceived academic achievement. This study reveals the underlying processes via which self-efficacy, time management, and psychological resilience contribute to perceived academic achievement among college students. The results of the study are thoroughly analyzed and discussed in the parts that follow.

The results supported H1 by demonstrating a strong positive link between college students perceived academic achievement and self-efficacy. This outcome is comparable with the results reported by Meng and Zhang (2023). Higher self-efficacy among students may increase their likelihood of use active coping strategies and maintain stronger learning motivation and persistence when facing academic difficulties. These characteristics may be associated with more positive perceptions of their academic achievement. This association may be especially important at the college level. Compared with students in basic education, college students receive less external supervision and face greater demands for autonomous learning. They need to set their own learning goals, manage the learning process, and deal with complex academic tasks. In this context, students' beliefs in their capabilities may serve as an important internal resource for maintaining learning engagement and overcoming difficulties. Students with higher self-efficacy may not only feel more confident in dealing with academic challenges but also be more willing to remain engaged in learning and develop positive learning habits (Basileo et al., 2024). This finding further supports SCT. The theory suggests that self-efficacy is related to individuals' goal choices, effort, and behavioral persistence, as well as corresponding behavioral outcomes (Bandura, 1986). This study extends this theoretical logic to the link between general self-efficacy and perceived academic achievement. The results show that beliefs about one's capabilities across different contexts are also associated with college students' positive evaluations of their academic performance. Thus, universities can provide students with opportunities for gradual success, positive feedback, and appropriate learning support to strengthen their self-efficacy. Such support may help students maintain learning engagement and develop more positive academic experiences.

Time management mediates the link between self-efficacy and perceived academic achievement, supporting H2. Students with higher self-efficacy perform better academically by enhancing their time management abilities. This result is in agreement with Ersoy and Peker (2020), which demonstrated that students with higher self-efficacy display significant executive functioning and self-regulation when faced with academic tasks. These students display greater self-discipline and proactivity in time management, allowing them to allocate their study time effectively and enhance learning efficiency. College students often need to manage various demands, including coursework, examinations, practical activities, and social life, while receiving relatively limited direct supervision over their daily learning schedules. In this context, students with higher self-efficacy may be more willing to invest time and effort in academic goals and to develop and follow learning plans. Effective time management may help them coordinate different tasks, reduce procrastination and task accumulation, and strengthen their sense of control over the learning process. These factors may be associated with higher perceived academic achievement (Ali et al., 2024; Dong et al., 2024). This result further clarifies the link between cognitive beliefs and academic outcomes proposed by SCT. It suggests that time management is an important self-regulatory mechanism that links college students' self-efficacy to effective learning behaviors (Bandura, 1986). Thus, universities can provide time management training in areas such as study planning, task prioritization, and progress monitoring. Such training may help college students translate positive beliefs about their capabilities into more organized and effective learning behaviors.

Psychological resilience mediates the link between self-efficacy and perceived academic achievement, supporting H3. This result is in agreement with the findings of Qin et al. (2023), which demonstrated that students with high self-efficacy typically display greater confidence and determination when encountering academic challenges. These students firmly believe in their ability to overcome difficulties, a belief that significantly strengthens their psychological resilience. As college students adjust to their academic programs, cope with examination pressure, and deal with academic setbacks, they may often face fluctuations in performance, task failure, and uncertainty about their future development. In these situations, students with higher self-efficacy might be prone to view difficulties as doable tasks instead of insurmountable dangers. This positive appraisal could be linked to increased psychological resilience (Qin et al., 2023). Students with greater psychological resilience may be better able to recover emotionally after experiencing stress or failure, adjust their learning strategies, and remain engaged in their studies. As a result, temporary setbacks may be less likely to be associated with persistently negative evaluations of their academic performance (Garcia-Martinez et al., 2022). This result extends the explanation of how self-efficacy operates within SCT. It indicates that self-efficacy is linked to perceived academic achievement through psychological resilience as an adaptive psychological resource (Bandura, 1986). Thus, universities can provide coping-skills training, stress management programs, and psychological support services to strengthen college students' psychological resilience. Such support may help students maintain confidence, regain engagement, and develop more positive academic experiences when facing academic difficulties.

The link between self-efficacy and perceived academic achievement is mediated by time management and psychological resilience, which supports H4. This result is in agreement with the findings of Antoniadou and Antoniadis (2024), who reported that better time management was linked to both decreased academic stress and increased psychological resilience. This sequential association may be due to the high demands for autonomous learning among college students. First, students with higher self-efficacy can be prone to view difficult tasks as manageable challenges. This appraisal may be associated with less anticipatory anxiety and avoidance, as well as greater initiative in setting goals, organizing tasks, and monitoring progress (Basileo et al., 2024). Thus, self-efficacy may be associated with more effective time management through stronger self-regulation. Second, time management is not only a learning strategy but also a behavioral resource. Effective time allocation may reduce task accumulation and feelings of losing control. It may also buffer academic stress and emotional exhaustion, thereby preserving the psychological resources needed to cope with difficulties (Trentepohl et al., 2022). These resources may allow students to adjust their psychological state and remain engaged in learning when facing academic setbacks. Thus, effective time management may be associated with greater psychological resilience and more positive perceived academic achievement (Li et al., 2023). According to conservation of resources theory, people aim to obtain and safeguard resources that support goal attainment. In this sequential pathway, students' use of time management as a behavioral resource may be supported by self-efficacy, a cognitive resource. Effective time management may then protect and build psychological resources associated with greater psychological resilience (Hobfoll, 1989). Thus, this pathway represents a sequential process in which self-efficacy is linked to psychological resilience through time management and is then linked with perceived academic achievement. From a resource transmission perspective, this finding extends the explanation offered by SCT regarding the relationships among cognitive beliefs, behavioral regulation, and academic outcomes. In practice, universities can provide integrated support for self-efficacy, time management, and coping with setbacks. Such support may contribute to more positive learning experiences and perceived academic achievement.

## Implications

### Theoretical implications

This research provides notable theoretical contributions to the discipline. First, drawing on SCT, this study suggests and tests a serial mediation model to systematically investigate the associations among self-efficacy, time management, psychological resilience, and perceived academic achievement. By utilizing this model, the study enhances the theoretical framework regarding the factors that relate to perceived academic achievement within educational psychology. Using PLS-SEM, the study reveals the complex interactions between self-efficacy, time management, and psychological resilience, and for the first time, it introduces the chain mediation roles of time management and resilience between self-efficacy and perceived academic achievement. This result enriches our understanding of how different factors are associated with perceived academic achievement in college students‘ learning processes, and offers valuable theoretical support for future research in related areas. Secondly, this study explores in depth the mediating roles of time management and psychological resilience between self-efficacy and perceived academic achievement. Specifically, time management is directly associated with perceived academic achievement and improves academic performance indirectly by boosting students' psychological resilience. This discovery broadens our perspective for academic research and expands the understanding of the mechanisms through which self-efficacy, time management, and psychological resilience function in academic learning.

### Practical implications

In addition to strengthening educational psychology's theoretical contributions, this study provides useful advice for raising college students' academic achievement, especially in the areas of boosting self-efficacy, time management, and psychological resilience. The study offers practical recommendations for educators and policymakers. First, educators should focus on helping students set learning goals that are both challenging and achievable, and enhance students' self-efficacy through timely positive feedback. Moreover, by presenting successful academic examples and role models, teachers can foster students' confidence and motivate them to tackle learning challenges more proactively. Secondly, in the teaching process, educators should teach effective time management techniques tailored to students' learning needs and contexts, such as time-blocking, task prioritization, and daily study checklists, to help students improve learning efficiency and, consequently, academic performance. Additionally, teachers should pay attention to developing students' psychological resilience by encouraging the use of positive emotional regulation strategies (e.g., meditation, deep breathing) to cope with learning stress, thus strengthening their ability to adapt to challenges and boosting their confidence in problem-solving.

## Limitations and future research

First, examining the long-term, dynamic correlations between the variables is limited by the study's cross-sectional approach. This design can only reveal the links between self-efficacy, time management, psychological resilience, and perceived academic achievement, without capturing the trends and interactions of these variables over time. To further investigate the dynamic evolution of these variables, future studies could consider adopting a longitudinal design, using multiple data measurements to observe changes in each variable at different time points, thereby providing a stronger theoretical basis for long-term educational interventions. Second, relying on self-report data may introduce social desirability bias and subjective response bias, potentially reducing the objectivity and accuracy of the findings. To improve the reliability of the study's findings, future research should incorporate other objective data collection methods, such as actual assessments, third-party evaluations, or tracking of learning behaviors. These data sources can help mitigate the biases linked to self-reports and improve the research findings' external validity. Third, the sample had an uneven demographic makeup and was selected from just six universities in Anhui Province. Nearly 70% of the participants were first-year students, and approximately 70% were female. This distribution may reflect structural bias in participant recruitment and questionnaire completion. Therefore, the sample could not adequately reflect the larger undergraduate population. When extrapolating the results to college students in other areas or with differing year level and gender distributions, care must be taken. Future studies could recruit participants from different regions and types of universities. They could also use stratified or quota sampling to achieve a more balanced distribution across gender and year level, thereby increasing sample representativeness and the generalizability of the findings. Finally, this study assessed perceived academic achievement using a self-report scale, which captures students' subjective evaluations of their academic performance and learning outcomes rather than objective achievement indicators such as course grades or grade point averages. Therefore, the findings may not fully reflect students' objective academic achievement. Future research could combine perceived academic achievement with course grades, grade point averages, or teacher evaluations to examine whether the findings remain consistent across different measures of academic achievement.

## Conclusion

In order to provide a better understanding of the complex mechanisms via which self-efficacy links to perceived academic achievement, this study investigates the chain mediation function of time management and psychological resilience. The results show that self-efficacy significantly and positively link to perceived academic achievement. By constructing a chain mediation model, this study verifies that self-efficacy directly links with perceived academic achievement and indirectly promotes improvements in perceived academic achievement by enhancing time management and psychological resilience. Specifically, the enhancement of self-efficacy enables students to plan their time more effectively, adopt coping strategies, and strengthen psychological resilience, thereby boosting their perceived academic achievement. This finding offers important guidance for educational practice, highlights the crucial role of improving students' self-efficacy, time management, and resilience in fostering perceived academic achievement. Educators should help students develop positive learning beliefs, set clear goals, and improve self-discipline and psychological resilience to better handle academic problems. The limitations of this study include its cross-sectional design, dependence on self-report data, and the inability to fully capture the multidimensional nature of perceived academic achievement. Future research should utilize longitudinal designs, combine objective data, and use multidimensional assessments of perceived academic achievement to further validate the findings and enhance their comprehensiveness.

## Data Availability

The raw data supporting the conclusions of this article will be made available by the authors, without undue reservation.
